# Joint Effect of Early Microvascular Damage in the Eye & Kidney on Risk of Cardiovascular Events

**DOI:** 10.1038/srep27442

**Published:** 2016-06-08

**Authors:** Wanfen Yip, Charumathi Sabanayagam, Peng Guan Ong, Uptal D Patel, Khuan Yew Chow, E Shyong Tai, Lieng H Ling, Tien Yin Wong, Carol Yim-lui Cheung

**Affiliations:** 1 Singapore Eye Research Institute, Singapore National Eye Centre, Singapore; 2 Department of Ophthalmology, Yong Loo Lin School of Medicine, National University of Singapore, Singapore; 3 Ophthalmology and Visual Sciences Academic Clinical Programme, Duke-NUS Graduate Medical School, National University of Singapore, Singapore; 4 Duke Clinical Research Institute, Duke University, Durham, NC, USA; 5 National Registry of Diseases Office, Singapore; 6 Department of Medicine, Yong Loo Lin School of Medicine, National University of Singapore, Singapore; 7 Cardiac Department, National University Heart Centre, Singapore; 8 Department of Ophthalmology and Visual Sciences, The Chinese University of Hong Kong, Hong Kong

## Abstract

Microalbuminuria is associated with an increased risk of cardiovascular disease (CVD), but not all individuals require treatment. Retinal microvascular abnormalities and microalbuminuria reflect early systemic microvascular changes. We examined the joint effect of retinal abnormalities and microalbuminuria on CVD risk in an Asian cohort. We conducted a prospective, population-based study. Retinal abnormalities were defined as presence of retinopathy and/or retinal venular widening. Microalbuminuria was defined as urinary albumin: creatinine ratio between 30–300 mg/g. Incident CVD was defined as newly diagnosed clinical stroke, acute myocardial infarction or CVD death. Cox regression models were performed to determine the associations between retinal abnormalities and microalbuminuria with risk of CVD, while controlling for established risk factors. 3,496 participants (aged ≥ 40) were free of prevalent CVD. During the follow-up (5.8 years), 126 (3.60%) participants developed CVD. Persons presenting with both retinal abnormalities and microalbuminuria were 6.71 times (95% CI, 2.68, 16.79) as likely to have incident CVD compared with those without either abnormalities. There was a significant interaction effect between retinal abnormalities and microalbuminuria on incident CVD. Assessment of retinal abnormalities in patients with microalbuminuria may provide additional value in identifying persons at risk of developing CVD.

Microalbuminuria is an increasing well-recognized indicator of cardiovascular disease (CVD) risk, with many studies consistently demonstrating an association between microalbuminuria and incident CVD^1^,^2^,^3^. However, microalbuminuria measurements can be highly variable^4^,^5^. For example, short-term hyperglycemia, exercise, urinary tract infections and acute febrile illness can cause transient elevations in urinary albumin excretion^4^,^6^. As such, microalbuminuria may result in an “overestimation” of CVD risk.

Retinal vessels, measuring 100 to 300 μm in size, offer a unique and easily accessible “window” to study the health and disease of the human microcirculation^7^,^8^. There is increasing evidence showing that retinal microvascular changes, reflecting systemic microcirculation damage, may provide additional value in CVD risk prediction^9^,^10^. Specifically, two retinal microvascular abnormalities, retinopathy and retinal venular caliber widening, measured from digital retinal photographs, have been found to be associated with subclinical CVD^11^,^12^,^13^,^14^ and to predict clinical CVD events^10^,^12^,^15^.

Because both microalbuminuria and retinal microvascular abnormalities are potential microvascular markers of CVD events, it may be possible that a “multiple markers” approach may be useful for risk stratification and identification of patients who are at higher risk of developing CVD. Measurement of retinal microvascular changes have been shown to be accurate and repeatable^7^ and retinal vascular imaging utilized as an adjunctive test have been shown to increase the precision of a more widely accepted test to benefit a specific subgroup of patients^16^ For example, Baumann *et al.* have recently reported that a combination of retinal arteriolar narrowing and albuminuria increased the risk of chronic kidney disease as compared with person who has no evidence of microvascular damage^17^.

In the present study, we aimed to examine the joint effect of retinal microvascular abnormalities and microalbuminuria on CVD risk in a multi-ethnic Asian cohort.

## Results

A total of 3,496 participants were free of prevalent CVD events at baseline. During a median follow-up of 5.8 years (baseline examination [2003 to 2007] and 31 December 2011), 126 (3.6%) participants developed CVD events. Of the CVD events, there were 53 (42.1%) AMI events, 38 (30.2%) stroke events and 35 (27.7%) CVD deaths. Table 1 shows the baseline characteristics of those who developed CVD events and those who did not.

Tables 2 shows the Cox proportional-hazards regression models of retinal arteriolar caliber, retinal venular caliber, retinopathy and microalbuminuria with CVD risk. Retinal venular widening (per SD increase: HR 1.33, 95% CI, 1.09 to 1.62) was independently associated with increased risk of CVD, after adjusting for age, gender, ethnicity, current smoking, diabetes, total cholesterol, HDL cholesterol, systolic BP, anti-hypertensive medication, eGFR, CRAE and retinopathy. The association was similar when retinal vascular caliber was analyzed as quartiles (HR 2.07; 95% CI, 1.15 to 3.73). The presence of retinopathy (HR 2.05, 95% CI, 1.30 to 3.22) was also significantly associated with increased risk of CVD. No significant associations were observed between retinal arteriolar caliber with incident CVD events. The presence of microalbuminuria was independently associated with increased risk of CVD events (HR 1.68, 95% CI, 1.13, 2.50), after adjusting for age, gender, ethnicity, current smoking, diabetes, total cholesterol, HDL cholesterol, systolic BP, anti-hypertensive medication, eGFR, CRVE, and retinopathy. In the supplementary analysis, further adjustment for hsCRP level in the multivariate models did not alter the associations between widened retinal venular caliber (HR 2.05, 95% CI, 1.12, 3.74), (per SD increase: 1.33, 95% CI, 1.09, 1.63), presence of retinopathy (HR 1.81, 95% CI: 1.12 to 2.92), and presence of microalbuminuria (HR 1.72, 95% CI, 1.15, 2.57) with incident CVD (Supplementary Table 1).

**Figure 1:**
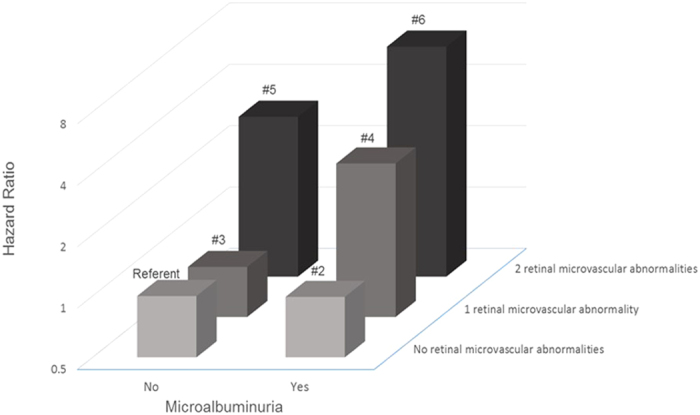
Joint effect of retinal microvascular abnormalities and microalbuminuria on risk of cardiovascular event. P-interaction for retinal microvascular abnormalities and microalbuminuria = 0.025 Group 1 (Referent): No retinal microvascular abnormalities and no microalbuminuria Group 2: Presence of microalbuminuria only Group 3: One retinal microvascular abnormality (either retinal venular widening or retinopathy) only Group 4: One retinal microvascular abnormality and presence of microalbuminuria Group 5: Two retinal microvascular abnormalities (retinal venular widening and retinopathy) and no presence of microalbuminuria Group 6: Two retinal microvascular abnormalities and presence of microalbuminuria.

The joint effect of microalbuminuria and retinal microvascular abnormalities (widened retinal venules and presence of retinopathy) on the risk of CVD events is graphically depicted in Fig. 1. Compared with the referent group, those with any retinal microvascular abnormalities and microalbuminuria (Group 4) were 2.83 times (95% CI, 1.65, 4.87) as likely to develop incident CVD. Persons presenting with both widened retinal venules and retinopathy but without microalbuminuria (Group 5) at baseline, were 3.04 times (95% CI, 1.29, 7.17) as likely to develop incident CVD, compared to those with neither retinal microvascular abnormalities nor microalbuminuria (referent). Importantly, persons presenting with the coexistent of widened retinal venules, retinopathy, and microalbuminuria (Group 6) were 6.71 times (95% CI, 2.68, 16.79) as likely to have incident CVD compared to persons without the presence of microalbuminuria and retinal microvascular abnormalities (Table 3). There was a significant interaction effect between retinal microvascular abnormalities and microalbuminuria on incident CVD (p-interaction = 0.025), demonstrating the association between retinal microvascular abnormalities with incident CVD was stronger in participants with microalbuminuria. In the supplementary analysis, we separately explored the joint effect of presence of clinical retinopathy and microalbuminuria on the risk of CVD events. We observed that individuals with microalbuminuria and clinical retinopathy were 3.69 times (HR: 3.69, 95% CI 2.00, 6.81) as likely to develop incident CVD, compared to those with neither presence of retinopathy nor microalbuminuria (referent) (Supplementary Fig. 1).

## Discussion

In this multi-ethnic Asian study, we found that persons with microvascular changes in the eye (retinal venular widening and/or presence of retinopathy) or in the kidney (manifesting as microalbuminuria) were at higher risk of CVD than those without these abnormalities. Importantly, we observed a significant joint effect of retinal microvascular abnormalities and microalbuminuria on the risk of CVD. Persons with evidence of microvascular damage in both the eyes and kidney were nearly 7 times as likely to develop CVD as those without these changes. Our findings suggest that assessment of retinal vasculature may be utilized as an adjunctive test to further identify persons who are at higher risk of CVD, in addition to the assessment of microalbuminuria and traditional risk factors.

We demonstrated that individually, the presence of retinal microvascular abnormalities and microalbuminuria are predictive of CVD events, consistent with current understanding and prior studies. The association between retinal microvascular abnormalities (widened retinal venules and retinopathy) and incident CVD has been previously reported in the general population and in specific populations such as in persons with type 2 diabetes^18^ and in patients with chronic kidney disease^19^. While these associations have also been observed in several population-based studies^10^,^20^, eGFR was not adjusted for in the multivariable models. This a major limitation in those population-based studies since eGFR is an important modifiable risk factor for CVD events^21^. In our study, we observed an independent association between retinal abnormalities (retinal venular widening and retinopathy) and incident CVD after adjusting for both diabetic status as well as eGFR level. Our study adds to the current literature by demonstrating that retinal microvascular abnormalities are indeed predictors of incident CVD, independent of eGFR and other traditional risk factors. A few possible reasons may explain this. First, retinal venular widening have been reported to be associated with inflammation and endothelial dysfunction^22^,^23^, which are important risk factors of cardiovascular disease (CVD)^24^. Consistently, it was reported in the Rotterdam Study^25^, the Beaver Dam Eye Study^26^, the Singapore Prospective Study Program^27^ and the Multi-Ethnic Study of Atherosclerosis^28^ that larger venular caliber was associated with inflammatory markers, including C-reactive protein (CRP) and interleukin-6 concentrations. This suggests that the association between retinal venular widening and CVD event may be mediated through inflammatory processes. Second, retinopathy signs (i.e. retinal exudates, haemorrhage) are pathogenically related to loss of pericytes, increased vascular permeability and retinal tissue ischemia, reflecting similar microvascular changes in other vascular beds^29^,^30^ Collectively, these lines of evidence support the concept that retinal microvasculature reflects cumulative microvascular damage that contributes to the progression of CVD. Several studies have also investigated the association between microalbuminuria and incident CVD in high-risk populations such as diabetic and hypertensive cohorts^31^,^32^ as well as in the elderly population^33^. Our current findings, one of the few in Asians, are consistent with those in Caucasian populations^1^,^2^.

Importantly, we further reported that persons with coexistent retinal microvascular abnormalities (retinal venular widening and retinopathy) and microalbuminuria had the worst prognosis (close to 7 times as likely to develop CVD), compared with those without either markers of microvascular damage.

Our finding suggests that additional assessment of retinal microvascular abnormalities can further identify patients who are more at risk of developing CVD in future, in particular among people with microalbuminuria. Therefore, retinal vascular imaging, which is readily accessible in routine clinical practice, is useful for targeting a more specific subgroup of patients who could benefit from more intensive investigations or early treatment. Our current finding is consistent with one other study which was conducted in the U.S. (National Health and Nutrition Examination Survey [NHANES])^20^. The authors reported the joint effect of retinopathy and chronic kidney disease with mortality in a general population^20^. However, this study did not examine other abnormalities of retinal microvascular damage (e.g. generalized retinal venular widening and retinal arteriolar narrowing) and CVD risk.

While our study is observational, there is increasing evidence to support that microvascular changes in the eye and kidney predict the risk of CVD. It is known that microvascular abnormalities in the kidney and eye have overlapping etiologies and similar pathological pathway (for example: endothelial dysfunction, vascular inflammatory processes, and oxidative stress)^34^,^35^, leading to increased capillary permeability and transvascular leakages^36^. However, it has been reported that presence of microalbuminuria can be detected in seemingly healthy participants^37^ and also in participants with subclinical vascular damage in the kidneys^38^. As such, it has been hypothesized that variable amount of microalbuminuria reflects varying degrees of vascular function and thus a person’s inherent susceptibility to subsequent organ damage^37^. On the other hand, retinal microvascular abnormalities reflect greater generalized vascular dysfunction in CVD-related target organs such as the heart and the brain^10^,^12^,^15^. Altogether, it is possible that among participants presenting with microalbuminuria and jointly presenting with retinal microvascular abnormalities may have a more diffuse systemic microvascular damage and thus, greater susceptibility to increased risk of CVD. Future replication studies providing robust and consistent evidence, as well as additional data about potential long-term benefit for patient outcomes, are needed.

The strengths of our study include a large population-based sample, quantitative and masked evaluation of retinal vessel diameters, standardized definition of CVD events, and the availability of information on potential confounding factors. Several limitations of this study should be addressed. First, the significant number of participants excluded from the analysis due to missing data, and only a third of the participants had their urine albumin quantified in SiMES, could have resulted in a selection bias. Second, the relatively small number of CVD events limited our ability to perform stratified analyses to examine associations between microvascular damage with stroke and AMI separately. This will require longer follow-up to accrue more CVD end-points. Third, other biomarkers of inflammation (e.g. interleukin-6) were not measured, and we were therefore unable to extensively examine the role of inflammation. Future studies comparing the specific and combined roles of microvascular disease in respect to the role of other inflammatory mediators on CVD development may be warranted.

In conclusion, we have demonstrated that the presence of microvascular changes in the eye (widened retinal venules and presence of retinopathy) and kidney (microalbuminuria) independently predict the risk of CVD. Notably, the risk of CVD is significantly increased in people with coexistent retinal abnormalities and microalbuminuria. Our findings highlight the combined prognostic value of two readily accessible and quantifiable markers of microvascular disease.

## Methods

### Study population

We conducted a prospective, population-based study in a multi-ethnic Asian cohort to determine the relationship of retinal microvascular abnormalities and microalbuminuria on risk of CVD. We utilized data from the Singapore Prospective Study Program (SP2) and the Singapore Malay Eye (SiMES) study. Details of both study participants and methods have been described elsewhere^39^,^40^.

**Figure 2:**
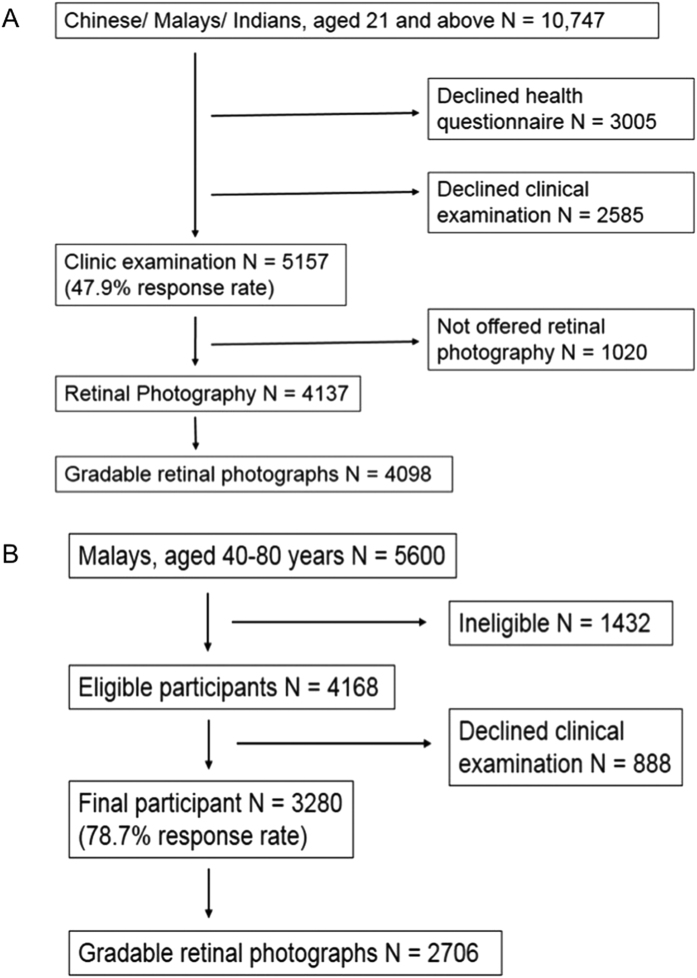
(**A**) Flow chart for selection and exclusion of individuals to form the final study population in SP2. (**B**) Flow chart for selection and exclusion of individuals to form the final study population in SiMES.

In brief, SP2 included participants from one of the four previous cross-sectional studies. The study sample was selected by the Ministry of Health, Singapore. From 2003 to 2007, 5157 participants attended the clinical examination and 4137 were offered retinal photography. Retinal photographs were available for 4098 participants (Fig. 2A). For the purposes of the current study, we excluded participants younger than 40 years of age, those with ungradable retinal photographs, missing microalbuminuria information, presence of macroalbuminura and with a history of CVD. Ethics approval was obtained from Institutional Review Boards of the National University of Singapore and Singapore General Hospital. Written informed consent was obtained from all participants.

SiMES is a population-based cross-sectional study examining eye diseases in urban Malay adults. In brief, the baseline examination was conducted from August 2004 through to June 2006, and included 3280 participants (Fig. 2B). We excluded participants with ungradable retinal photographs, missing microalbuminuria information, presence of macroalbuminura and with a history of CVD. Written, informed consent was obtained from each participant; the study conducted adhered to the Declaration of Helsinki. Ethical approval was obtained by the Singapore Eye Research Institute Institutional Review Board. Study methods were carried out in accordance with the approved guidelines.

### Retinal microvascular changes

Retinal fundus photographs of both eyes were taken after dilating the pupils with 1% tropicamide and 2.5% phenylephrine hydrochloride, using a digital non-mydriatic retinal camera (CR-DGi with a 10D SLR backing; Canon, Tokyo, Japan). Two retinal images of each eye were obtained, one centered on the optic disc and another centered on the fovea.

**Figure 3:**
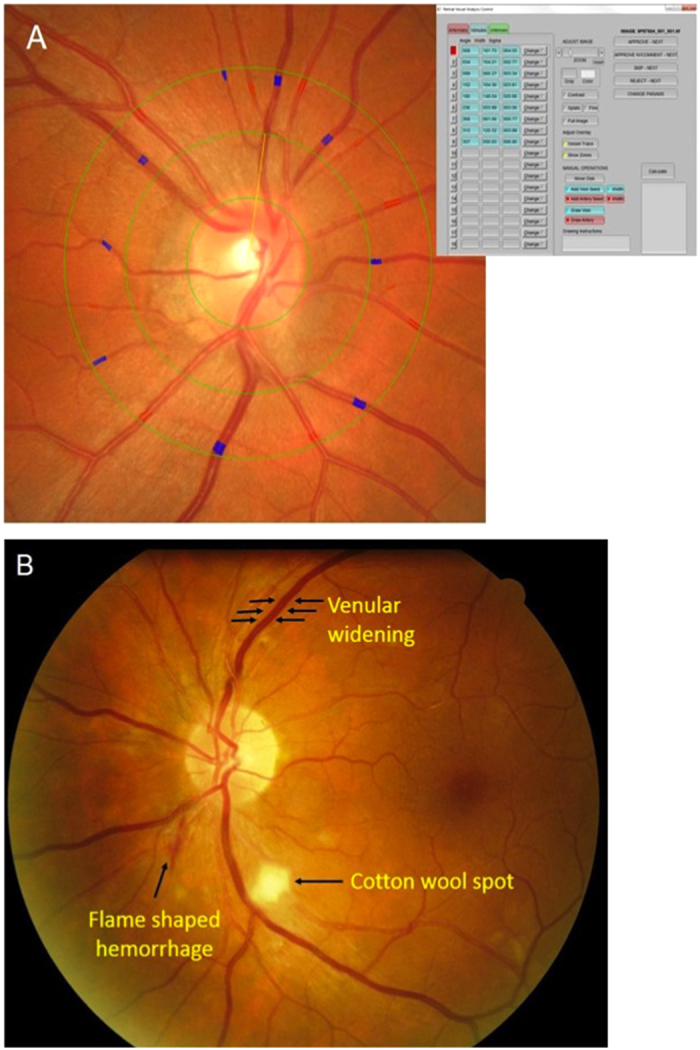
Examples of (**A**) IVAN interface for retinal caliber measurements; (**B**) retinopathy signs.

Trained graders, masked to the participants’ characteristics, performed retinal vascular caliber measurements using a computer-based program, Interactive Vessel Analysis software (IVAN) program (University of Wisconsin, Madison, US) (Fig. 3A)^41^. Retinal vascular caliber was measured through a specified zone of 0.5 to 1 disc diameter from the optic disc margin. Optic disc-centered image of the right eye were analyzed, and in those without gradable right eye images, left eye was analyzed. Based on the revised Knudtson-Parr-Hubbard formula^42^, the retinal arteriolar and venular calibers were summarized as central retinal arteriolar equivalent (CRAE) and central retinal venular equivalent (CRVE), respectively.

Two hundred randomly selected retinal photographs were re-graded by the same grader to assess intra-grader reliability (intraclass correlation coefficients [95% confidence interval (CI)] 0.99 (0.98–0.99) for CRAE and 0.94 (0.92–0.96) for CRVE)^39^.

Retinopathy was considered present if any characteristic lesions (microaneurysms, haemorrhages, cotton wool spots, intraretinal microvascular abnormalities, hard exudates, venous beading and new vessels) were found (Fig. 3B)^43^. For each eye, a retinopathy severity score was assigned accordingly and retinopathy (or clinical retinopathy) was defined as being present if the retinopathy score (a scale modified from the Airlie House classification system) was at level 15 or higher^43^.

### Microalbuminuria

Spot untimed urine samples were collected for measurement of albumin and creatinine. Albumin was measured in mg/L and creatinine in mmol/L. The concentration ratio of urine albumin to creatinine expressed in mg/g was used to estimate the total daily albumin excretion. Presence of microalbuminuria was defined as a urinary albumin: creatinine ratio (ACR) of between 30–300 mg/g based on the National Kidney Foundation’s Kidney Disease Outcome Quality Initiative working group definition^44^.

### Assessment of cardiovascular disease events

History of stroke/AMI events was ascertained through self-reporting and/or CVD event documented by National Registry of Diseases Office (NRDO), Singapore.

Incident CVD event was defined as newly diagnosed clinical stroke or acute myocardial infarction (AMI) or CVD death documented by NRDO that occurred during the period between baseline examination and 31 December, 2011, obtained by linking with the stroke, AMI cases and CVD deaths registered by NRDO by record linkage.

MI was defined as either (1) definitive or clinical MI; (2) death cases signed up by pathologists or physicians as MI, with or without necropsy done. Incident MI was identified by linkage with the Singapore Myocardial Infarction Registry (SMIR), a nation-wide registry under the purview of the National Registry of Diseases Office (NRDO). The epidemiological data in SMIR included all MI diagnosed and coded with International Classification of Diseases 9^th^ revision (ICD-9) 410 in all restructured hospital^45^. Because the Register is electronically captured and is compulsory by law, and because of a unique identifier number for all Singapore citizens and residents (National Registration Identity Card), the Registry is expected to provide good coverage for AMI cases.

Stroke data was obtained from the Singapore Stroke Registry gathered from Mediclaims and case funding from restructured hospitals electronic medical records using ICD-9 codes 430 to 434 and 436 to 437, while excluding 432.1, 435 and 438. Data capture is estimated at 94% since private hospitals were not included^46^. CVD mortality was defined as CVD as the primary cause of death as stated on the death certificate.

### Other variables

Information on participants’ demographic characteristics and medical history was obtained by using a standardized questionnaire administered by trained personnel. Age was defined as the age at the time of clinic examination. Height was measured in cm using a wall-mounted measuring tape and weight was measured in kg using a digital scale. Body mass index (BMI) was calculated as body weight divided by height squared and expressed as kg/m^2^. Systolic and diastolic blood pressures (BP) were measured twice using a digital BP monitor (Dinamap model Pro Series DP110X-RW, 100V2, GE Medical Systems Information Technologies Inc, Milwaukee, WI). A third measurement was made if the systolic BP differed by >10 mm Hg or the diastolic BP differed by >5 mm Hg. The mean between the 2 closest readings was taken as the BP of that individual.

Venous blood samples were analyzed at the National University Hospital Referral Laboratory for biochemical testing of serum total cholesterol, high-density lipoprotein (HDL) cholesterol, low-density lipoprotein cholesterol, triglycerides, glycosylated hemoglobin (HbA1c), creatinine, glucose and high-sensitivity C-reactive protein (hsCRP). In SP2, diabetes mellitus was defined as fasting plasma glucose ≥7 mmol/L or HbA1c of ≥6.5% or self-reported physician-diagnosed diabetes or use of glucose-lowering medication; In SiMES, diabetes was defined as a casual plasma glucose measurement of ≥11.1 mmol/L or HbA1c of ≥6.5% or self-reported physician-diagnosed diabetes or use of glucose-lowering medication. The estimated glomerular filtration rate (eGFR) was estimated from serum creatinine using the recently developed Chronic Kidney Disease Epidemiology Collaboration (CKD-EPI) equation^47^.

### Statistical Analysis

All statistical analyses were performed using STATA statistical software (Version 12, StataCorp, College Station, Texas). The outcome of interest was incident CVD event and the exposures of interest were retinal vascular caliber (analyzed as quartiles), presence of retinopathy (analyzed as binary) and presence of microalbuminuria (analyzed as binary). Retinal arteriolar (CRAE) and venular (CRVE) calibers were also analyzed as continuous variables (per each standard deviation [SD] increase/decrease). As previous studies have shown that retinal arteriolar narrowing and retinal venular widening are associated with CVD, we used the widest quartile of retinal arteriolar caliber (quartile 4) as the referent category in the retinal arteriolar narrowing analysis, and the narrowest quartile of retinal venular caliber (quartile 1) as referent category in the retinal venular widening analysis^7^.

First, we performed Cox proportional-hazards regression analysis to examine the relations of CRAE, CRVE, retinopathy, microalbuminuria to the risk of CVD, initially adjusted for age, gender and ethnicity, and additionally for smoking status, total cholesterol, HDL cholesterol, systolic BP, diabetic status, use of anti-hypertensive medication, eGFR and other retinal microvascular abnormalities at baseline. The proportional hazards assumption for each of these models was examined, and there was no evidence that this was violated. In supplementary analysis, we further adjusted for hsCRP level to examine whether the associations of CRAE, CRVE, retinopathy, microalbuminuria and incident CVD events were attenuated.

Second, we determined the joint effect of retinal microvascular abnormalities and microalbuminuria on the risk of CVD events. Retinal microvascular abnormalities were defined as presence of widened retinal venules (quartile 4) and/or retinopathy because these two parameters showed significant associations with incident CVD event in the Cox proportional-hazards regression analysis (Table 2). We categorized the population into 6 groups (i.e., Group 1 (Referent): no retinal microvascular abnormalities and no microalbuminuria; Group 2: no retinal microvascular abnormalities, and presence of microalbuminuria; Group 3: one retinal microvascular abnormality (either widened CRVE or retinopathy), but no microalbuminuria; Group 4: one retinal microvascular abnormality, and presence of microalbuminuria; Group 5: two retinal microvascular abnormalities (widened CRVE and retinopathy) and no presence of microalbuminuria; Group 6: two retinal microvascular abnormalities, and presence of microalbuminuria. We calculated the hazard ratios (HR) for each group vs. the referent group (Group 1) to the risk of incident CVD event. Separately, interaction between retinal microvascular abnormalities and microalbuminuria were evaluated by including a cross-product interaction term as an independent variable (i.e. retinal microvascular abnormalities × microalbuminuria).

In supplementary analysis, we separately explored the joint effect of presence of clinical retinopathy and microalbuminuria on the risk of CVD events.

## Additional Information

**How to cite this article**: Yip, W. *et al.* Joint Effect of Early Microvascular Damage in the Eye & Kidney on Risk of Cardiovascular Events. *Sci. Rep.* **6**, 27442; doi: 10.1038/srep27442 (2016).

## Supplementary Material

Supplementary Information

## Figures and Tables

**Table 1:** Baseline characteristics of participants who did not develop incident CVD and those who developed incident CVD.

	No CVD events (N = 3370)	CVD events (N = 126)	p-value
	Mean (SD)/N (%)	Mean (SD)/N (%)	
Age, years	52.93 (8.89)	61.45 (11.72)	<0.001
Ethnicity (Chinese/Malay/Indian)	1625 (48.22)/1249 (37.06)/496 (14.72)	37 (29.37)/61 (48.41)/28 (22.22)	<0.001
Gender, male	1603 (47.57)	97 (76.98)	<0.001
Body mass index, kg/m^2^	24.74 (4.54)	25.21 (4.49)	0.257
Systolic blood pressure, mmHg	135.85 (20.96)	153.83 (25.73)	<0.001
Diastolic blood pressure, mmHg	79.17 (10.69)	85.60 (13.07)	<0.001
Total cholesterol, mmol/L	5.37 (0.96)	5.57 (1.15)	0.023
HDL cholesterol, mmol/L	1.39 (0.34)	1.22 (0.27)	<0.001
LDL cholesterol, mmol/L	3.41 (0.92)	3.50 (0.72)	0.524
Estimate glomerular filtration rate, mL/min/1.73 m^2^	83.38 (16.87)	72.41 (17.37)	<0.001
High sensitivity C-reactive protein, mg/L	3.00 (6.52)	3.50 (7.51)	0.416
Microalbuminuria, yes	460 (13.65)	45 (35.71)	<0.001
Diabetes, yes	1099 (32.61)	57 (45.24)	0.003
Hypertension, yes	1576 (46.77)	92 (73.02)	<0.001
Current smoking, yes	577 (14.15)	42 (33.33)	<0.001
Retinal arteriolar caliber, μm	141.66 (14.54)	137.59 (15.78)	0.002
Retinal venular caliber, μm	219.80 (20.67)	224.16 (21.0)	0.002
Any retinopathy, yes	285 (8.37)	25 (19.84)	<0.001

*p-value for differences between subjects who did not develop incident CVD and those who developed incident CVD, by t-test or chi-square test as appropriate.

**Table 2:** Relation of retinal vascular calibers, presence of retinopathy and presence of microalbuminuria on risk of future cardiovascular disease event.

	No. at risk	Incident cases, %	HR (95% CI)*	HR (95% CI)†
Retinal arteriolar caliber (μm)				
Quartile 4 (151.08 − 206.31)	876	28 (3.20)	Referent	Referent
Quartile 3 (141.72 − 151.06)	873	18 (2.06)	0.63 (0.35, 1.15)	0.75 (0.41, 1.38)
Quartile 2 (132.08 − 141.71)	873	28 (3.21)	0.82 (0.48, 1.38)	1.03 (0.59, 1.80)
Quartile 1 (71.68 − 32.07)	874	52 (5.95)	1.03 (0.71, 1.80)	1.50 (0.87, 2.58)
P for trend			0.328	0.071
per SD decrease (14.60)	3496	126 (3.60)	1.05 (0.89, 1.24)	1.20 (0.98, 1.47)
Retinal venular caliber (μm)				
Quartile 1 (104.03−206.34)	874	24 (2.75)	Referent	Referent
Quartile 2 (206.37 − 220.11)	874	27 (3.09)	1.33 (0.77, 2.31)	1.23 (0.69, 2.19)
Quartile 3 (220.12 − 233.33)	874	33 (3.78)	**1.05 (1.01, 3.02)**	1.64 (0.93, 2.89)
Quartile 4 (233.34 − 296.67)	874	42 (4.81)	**2.25 (1.35, 3.76)**	**2.07 (1.15, 3.73)**
P for trend			**0.001**	**0.008**
per SD increase (20.70)	3496	126 (3.60)	**1.32 (1.13, 1.55)**	**1.33 (1.09, 1.62)**
Presence of Retinopathy				
No	3189	101 (3.17)	Referent	Referent
Yes	307	25 (8.14)	**2.20 (1.42, 3.41)**	**2.05 (1.30, 3.22)**
	**No. at risk**	**Incident cases, %**	HR (95% CI)*	**HR (95% CI)‡**
Microalbuminuria				
No	2,991	81 (2.71)	Referent	Referent
Yes	505	45 (8.91)	**2.27 (1.57, 3.32)**	**1.68 (1.13, 2.50)**

^*^Adjusted for age, gender and ethnicity.

^†^Further adjusted for current smoking, diabetes, total cholesterol, HDL cholesterol, systolic blood pressure, anti-hypertensive medication, eGFR, retinal arteriolar caliber (when the main exposure is retinal venular caliber and vice versa) and retinopathy.

^‡^Further adjusted for current smoking, diabetes, total cholesterol, HDL cholesterol, systolic blood pressure, anti-hypertensive medication, eGFR, retinal venular caliber and retinopathy. HR: Hazard Ratio.

**Table 3:** Joint effect of retinal microvascular abnormalities and microalbuminuria on risk of future cardiovascular disease event.

Group	Retinal Microvascular Abnormalities	Microalbuminuria	No. at risk	No. of incident cases (%)	HR (95% CI)*
1	0	0	2098	54 (2.57)	Referent
2	0	1	312	17 (5.54)	0.99 (0.56, 1.77)
3	1	0	821	21 (2.56)	0.88 (0.51, 1.50)
4	1	1	170	22 (12.94)	**2.83 (1.65, 4.87)**
5	2	0	72	6 (8.33)	**3.04 (1.29, 7.17)**
6	2	1	23	6 (26.09)	**6.71 (2.68, 16.79)**

^*^Adjusted for age, gender, ethnicity, current smoking, diabetes, total cholesterol, HDL cholesterol, systolic blood pressure, anti-hypertensive medication, eGFR.
