# Supplementary material for: Joint Effect of Early Microvascular Damage in the Eye & Kidney on Risk of Cardiovascular Events
Source: Sci Rep. 2016 Jun 8;6:27442. doi: 10.1038/srep27442 (PMC4897605; doi:10.1038/srep27442)
Supplement: Supplementary Information [file srep27442-s1.doc]

**Joint Effect of Early Microvascular Damage in the Eye & Kidney on Risk of Cardiovascular Events**

Wanfen Yip, BSc (Hons),^1,2^, Charumathi Sabanayagam, MD, PhD,^1,2,3^, Peng Guan Ong, BSc (Hons),^1^, Uptal D. Patel, MD,^4^, Khuan Yew Chow, MBBCh,^5^, E Shyong Tai, MCRP,^6^ , Lieng H. Ling, MD, ^6,7^, Tien Yin Wong, MD, PhD, ^1,2,3^,Carol Yim-lui Cheung*, PhD, ^1,2,3,8^

1. Singapore Eye Research Institute, Singapore National Eye Centre, Singapore
2. Department of Ophthalmology, Yong Loo Lin School of Medicine, National University of Singapore, Singapore
3. Ophthalmology and Visual Sciences Academic Clinical Programme, Duke-NUS Graduate Medical School, National University of Singapore, Singapore
4. Duke Clinical Research Institute, Duke University, Durham, NC, US
5. National Registry of Diseases Office, Singapore
6. Department of Medicine, Yong Loo Lin School of Medicine, National University of Singapore
7. Cardiac Department, National University Heart Centre, Singapore
8. Department of Ophthalmology and Visual Sciences, The Chinese University of Hong Kong, Hong Kong

**Supplementary Table/ Figure**

| **Supplementary Table 1.** Relation of retinal vascular parameters, presence of retinopathy and presence of microalbuminuria on risk of future cardiovascular disease event | | | |
| --- | --- | --- | --- |
|  | **No. at risk** | **Incident cases, %** | **HR (95% CI) ^*^** |
| **Retinal arteriolar caliber** (µm) |  |  |  |
| Quartile 4 ( 151.08 - 206.31) | 876 | 28 (3.20) | Referent |
| Quartile 3 ( 141.72 -151.06) | 873 | 18 (2.06) | 0.76 ( 0.41, 1.40) |
| Quartile 2 ( 132.08 - 141.71) | 873 | 28 (3.21) | 0.99 (0.56, 1.76) |
| Quartile 1 ( 71.68 - 32.07) | 874 | 52 (5.95) | 1.42 (0.81, 2.47) |
| P for trend |  |  | 0.126 |
| per SD decrease (14.60) | 3496 | 126 (3.60) | 1.18 (0.96, 1.45) |
| **Retinal venular caliber** (µm) |  |  |  |
| Quartile 1 (104.03 -206.34) | 874 | 24 (2.75) | Referent |
| Quartile 2 (206.37 - 220.11) | 874 | 27 (3.09) | 1.23 (0.69, 2.22) |
| Quartile 3 (220.12 - 233.33) | 874 | 33 (3.78) | 1.64 (0.92, 2.90) |
| Quartile 4 (233.34 - 296.67) | 874 | 42 (4.81) | **2.05 (1.12, 3.74)** |
| P for trend |  |  | **0.011** |
| per SD increase (20.70) | 3496 | 126 (3.60) | **1.33 (1.09, 1.63)** |
| **Presence of Retinopathy** |  |  |  |
| No | 3189 | 101 (3.17) | Referent |
| Yes | 307 | 25 (8.14) | **1.81 (1.12, 2.92)** |
|  | **No. at risk** | **Incident cases, %** | **HR (95% CI)** ^†^ |
| **Microalbuminuria** |  |  |  |
| No | 2,991 | 81 (2.71) | Referent |
| Yes | 505 | 45 (8.91) | **1.72 (1.15 to 2.57)** |
| *Adjusted for age, gender and ethnicity current smoking, diabetes, total cholesterol, HDL cholesterol, systolic blood pressure, and anti-hypertensive medication, eGFR, retinal arteriolar caliber (when the main exposure is retinal venular caliber and vice versa), retinopathy and hsCRP. ^†^ Adjusted for age, gender and ethnicity, current smoking, diabetes, total cholesterol, HDL cholesterol, systolic blood pressure, and anti-hypertensive medication, eGFR, retinal venular caliber retinopathy and hsCRP. | | | |
| HR: Hazard Ratio; hsCRP: high-sensitivity C-reactive protein | | | |

**Supplementary Figure 1.** Joint effect of clinical retinopathy and microalbuminuria on risk of cardiovascular event


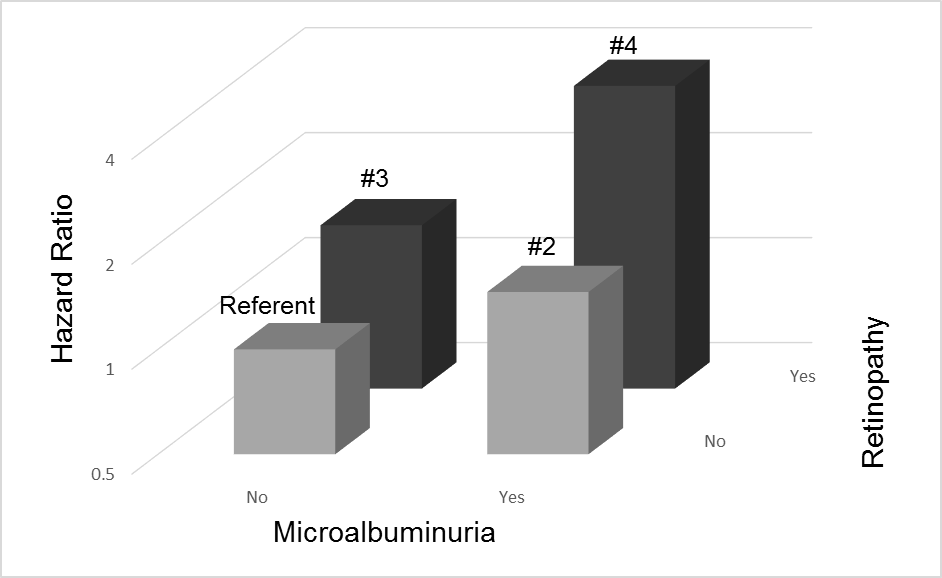


Group 1 (Referent): No retinopathy and no microalbuminuria

Group 2: Presence of microalbuminuria only

Group 3: Presence retinopathy only

Group 4: Presence of retinopathy and microalbuminuria
